# Cerebrovascular disease case identification in inpatient electronic medical record data using natural language processing

**DOI:** 10.1186/s40708-023-00203-w

**Published:** 2023-09-02

**Authors:** Jie Pan, Zilong Zhang, Steven Ray Peters, Shabnam Vatanpour, Robin L. Walker, Seungwon Lee, Elliot A. Martin, Hude Quan

**Affiliations:** 1 Centre for Health Informatics, Cumming School of Medicine, University of Calgary, Calgary, AB Canada; 2 Department of Community Health Sciences, Cumming School of Medicine, University of Calgary, Calgary, AB Canada; 3 Department of Clinical Neurosciences, Cumming School of Medicine, University of Calgary, Calgary, AB Canada; 4 Alberta Health Services, Edmonton, AB Canada

**Keywords:** Cerebrovascular disease, Machine learning, Natural language processing, Electronic health records, Disease identification

## Abstract

**Background:**

Abstracting cerebrovascular disease (CeVD) from inpatient electronic medical records (EMRs) through natural language processing (NLP) is pivotal for automated disease surveillance and improving patient outcomes. Existing methods rely on coders’ abstraction, which has time delays and under-coding issues. This study sought to develop an NLP-based method to detect CeVD using EMR clinical notes.

**Methods:**

CeVD status was confirmed through a chart review on randomly selected hospitalized patients who were 18 years or older and discharged from 3 hospitals in Calgary, Alberta, Canada, between January 1 and June 30, 2015. These patients’ chart data were linked to administrative discharge abstract database (DAD) and Sunrise^™^ Clinical Manager (SCM) EMR database records by Personal Health Number (a unique lifetime identifier) and admission date. We trained multiple natural language processing (NLP) predictive models by combining two clinical concept extraction methods and two supervised machine learning (ML) methods: random forest and XGBoost. Using chart review as the reference standard, we compared the model performances with those of the commonly applied International Classification of Diseases (ICD-10-CA) codes, on the metrics of sensitivity, specificity, positive predictive value (PPV), and negative predictive value (NPV).

**Result:**

Of the study sample (n = 3036), the prevalence of CeVD was 11.8% (n = 360); the median patient age was 63; and females accounted for 50.3% (n = 1528) based on chart data. Among 49 extracted clinical documents from the EMR, four document types were identified as the most influential text sources for identifying CeVD disease (“nursing transfer report,” “discharge summary,” “nursing notes,” and “inpatient consultation.”). The best performing NLP model was XGBoost, combining the Unified Medical Language System concepts extracted by cTAKES (e.g., top-ranked concepts, “Cerebrovascular accident” and “Transient ischemic attack”), and the term frequency-inverse document frequency vectorizer. Compared with ICD codes, the model achieved higher validity overall, such as sensitivity (25.0% vs 70.0%), specificity (99.3% vs 99.1%), PPV (82.6 vs. 87.8%), and NPV (90.8% vs 97.1%).

**Conclusion:**

The NLP algorithm developed in this study performed better than the ICD code algorithm in detecting CeVD. The NLP models could result in an automated EMR tool for identifying CeVD cases and be applied for future studies such as surveillance, and longitudinal studies.

**Supplementary Information:**

The online version contains supplementary material available at 10.1186/s40708-023-00203-w.

## Introduction

Accurate identification of patients with cerebrovascular diseases (CeVD) is important for health services research, surveillance and monitoring, risk adjustment, and quality improvement measurement [1, 2]. The standard approach to identify conditions is coded administrative hospital data using International Classification of Disease (ICD) terminology. Although structured codes are widely available and highly standardized, some conditions, including CeVD, are under-coded. Quan et al. [3] validated the ICD algorithms against chart review and reported a sensitivity of 46.3% for detecting CeVD diseases in both ICD-9 and ICD-10-CA. To overcome the shortcomings of ICD code-based algorithms, medical chart reviews act as a gold standard for case identification. Unfortunately, chart review is time- and resource-intensive requiring health professionals familiar with specific conditions [4, 5].

Electronic medical records (EMRs) are becoming increasingly popular for collecting health information [6], and can be used to improve the accuracy of identifying conditions such as CeVD. Among the components of EMR, free text notes contain detailed descriptions and give health professionals great flexibility to report conditions and comorbidities. Natural Language Processing (NLP) is an artificial intelligence technique to analyze human languages and retrieve clinically relevant information for detecting and predicting medical conditions [7]. A recent literature conducted by our team yielded few studies using NLP on clinical notes for patients with CeVD conditions [8]. Existing studies have focused on identifying ischemic stroke [9–11] and cerebral aneurysms [12], predicting the cerebrovascular causes of ischemia [13], and detecting complications of stroke [14]. Most previous studies focus on specific conditions within CeVD and have limited access to a complete set of clinical notes from EMRs, using only admission notes or radiology reports.

In this study, we explored all available types of inpatient clinical notes from an EMR to identify a broad spectrum of CeVD cases. The CeVD cases were defined by our previous ICD-10 algorithm [3]. We hypothesized that using NLP techniques on these clinical notes would better detect CeVD cases than ICD-based algorithms and existing ML algorithms with limited data source types.

## Methods

### Study population

In this retrospective cohort study, we randomly selected patients who were at least 18 years of age and discharged from three acute care facilities in Calgary, Canada, between January 1 and June 30, 2015. Obstetric admissions were excluded because they have a short length of stay and lack conditions of interest. We randomly selected one hospitalization per patient if multiple discharges occurred during the study period [15]. Six nurses reviewed charts to determine the existence of CeVD [15].

### Data sources

#### EMR: Sunrise Clinical Manager (SCM)

The EMR data are from SCM, a city-wide, population level EMR system used in the three acute care hospitals in Calgary. SCM provides patient-level clinical information containing medical and nursing orders, medication records, clinical documentation, diagnostic imaging and lab results [16].

#### Administrative Discharge Abstract Database: DAD

The inpatients’ administrative, clinical, and demographic information at the time of discharge is coded in the DAD [17]. The clinical coder records up to 25 diagnostics codes for each inpatient based on available information from patient charts. The DAD, EMR data and chart data were linked with Personal Health Number (a unique lifetime identifier), chart number (a distinctive number associated with a patient’s admission), and admission date.

### Phenotyping algorithm framework

**Fig. 1:**
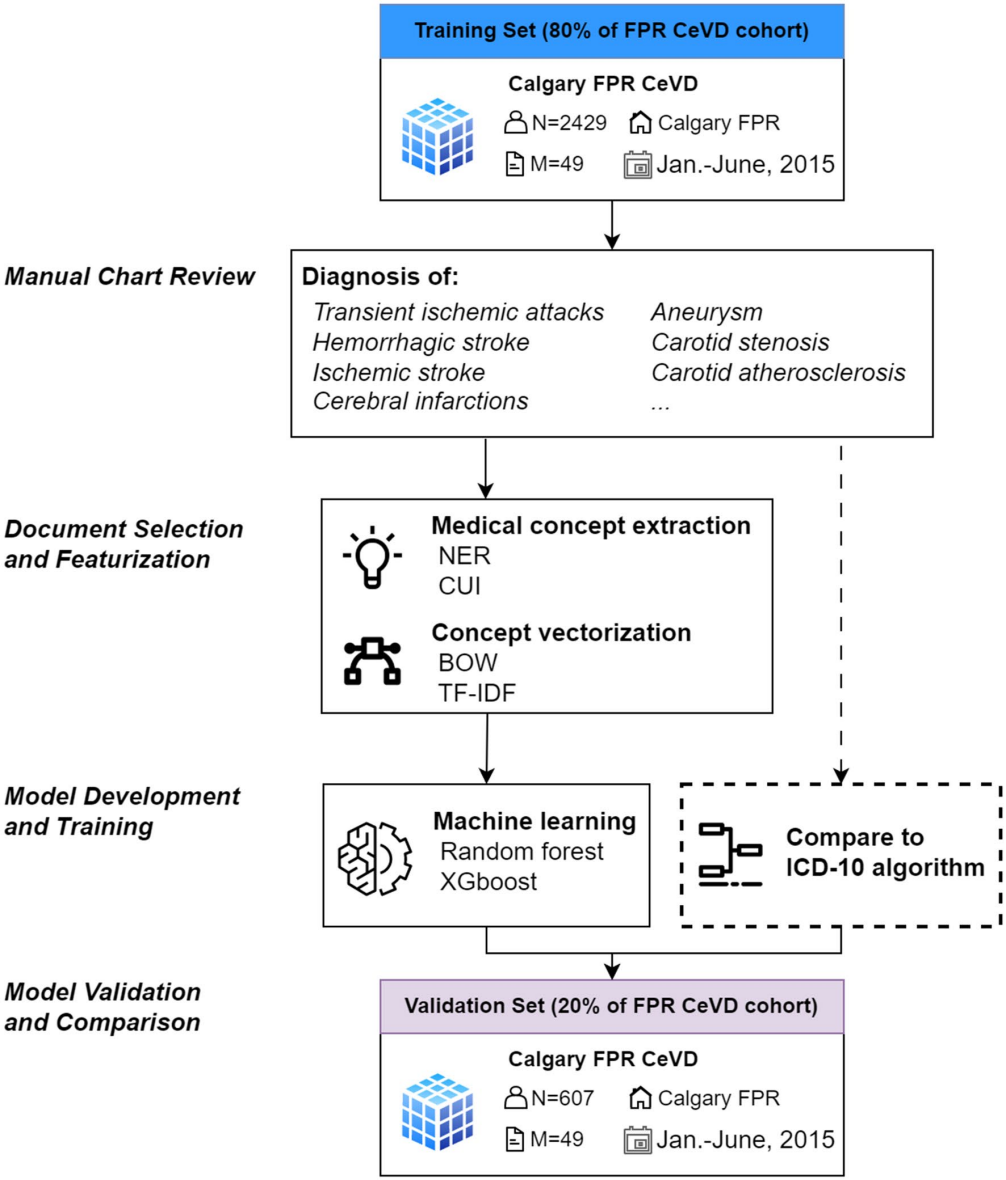
NLP-based CeVD detection framework using EMR data. It consists of manual chart review, data preprocessing, featurization, and model training, development, and validation. FPR represents three acute care facilities in Calgary, Foothills Medical Centre, Peter Lougheed Centre and Rockyview General Hospital; NER represents Named Entity Recognition (NER), a subtask of NLP that seeks to identify named objects from free-text; CUI represents Concept Unique Identifiers which map synonyms to a unique identifier; BOW represents bag of words; TF-IDF represents term frequency and inverse document frequency

We trained, validated, and tested an EMR data-driven phenotyping algorithm using NLP techniques to detect CeVD. NLP techniques are used to process and analyze human language, and contain a wide range of tasks, including named entity recognition (NER), information extraction, and text classification [11, 16]. They were applied to analyze the free text clinical notes and derive a CeVD phenotype to detect the disease automatically. As depicted in Fig. 1, the general framework consists of (1) input document selection from patients’ clinical notes, (2) model training, and (3) performance evaluation using chart review as a reference standard.

#### Document selection and feature engineering

Many types of clinical notes could be generated during the hospitalization of patients involved in this study, such as nursing transfer reports, inpatient consultations, discharge summaries, and surgical assessment and history. However, not all document types contribute equally to the detection of CeVD. Noise and redundant information can hamper the detection performance of ML models [18]. The first step is determining and selecting the appropriate document type(s) sensitive to CeVD identification.

**Fig. 2:**
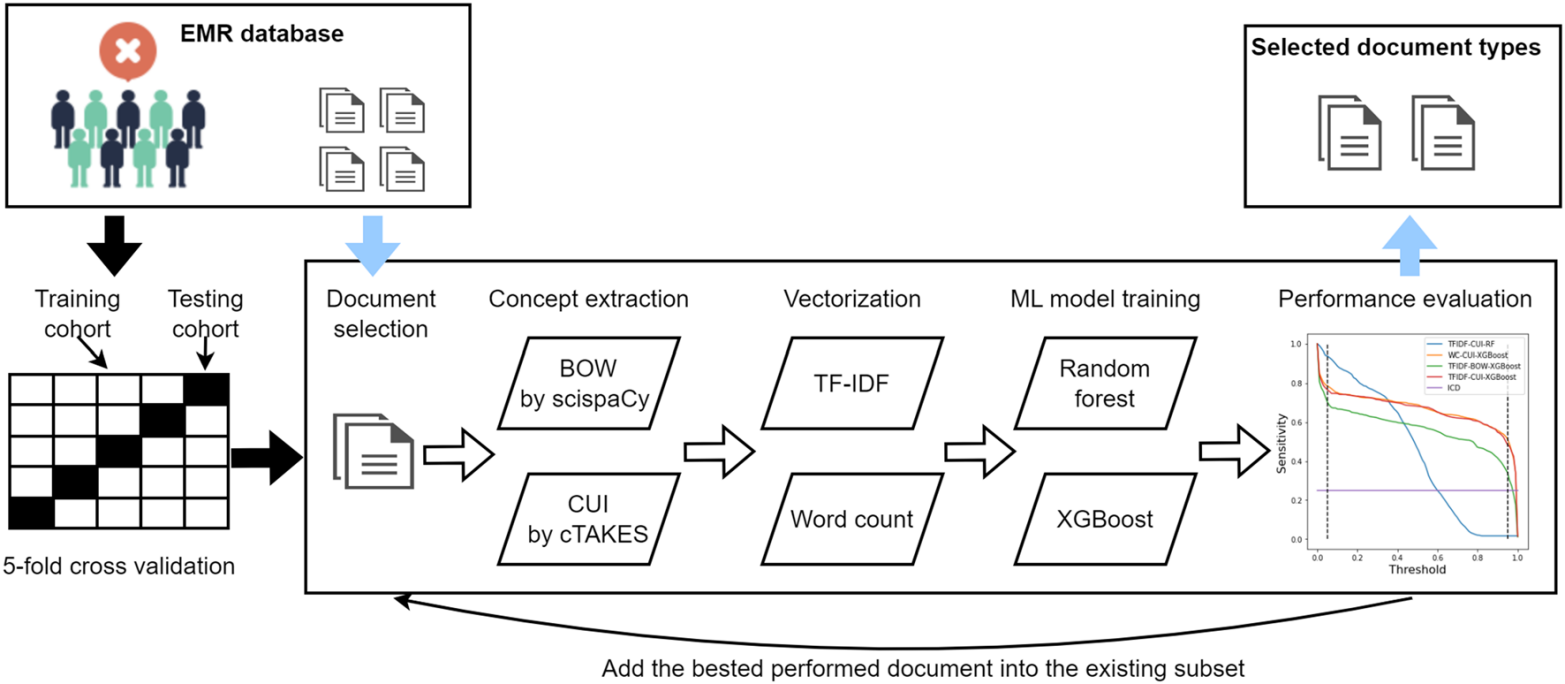
Document selection and featuring process based on the developed NLP models. All the document types are from 3036 patients’ clinical notes during hospitalization

The method we used is a feedforward sequential selection method [19], to iteratively add the document type that contributes most to model performance, until the performance stops increasing or reaches a predefined criterion, as shown in Fig. 2. All the documents are first converted into vectors by (1) extracting relevant medical concepts from the text and (2) turning concepts into numeric features [20]. To examine the extraction performance, we compared two types of commonly used concept extraction methods: Bag of Words (BOW) using ScispaCy [21] and Concept Unique Identifiers (CUIs) from the Unified Medical Language System using cTAKES (see Additional file 1 for a detailed explanation) [22]. We also compared two types of feature construction methods: Term Frequency-Inverse Document Frequency (TF-IDF) and word count. The obtained vectors are fed into the ML models and validated by the model performance. To estimate better generalization of the selected document types, fivefold cross validation was applied to the selected patients (i.e., 80% training, n = 2429 and 20% test, n = 607). The model development is detailed in the following section.

#### Model development

The model outcome is a binary classification where hospitalized patients with CeVD are considered positive cases. Two supervised ML methods were trained, validated, and tested using the obtained input vectors and chart review output labels, including random forest (RF) and XGBoost [23, 24]. The two methods are known for handling datasets with high dimensionality, missing data and outliers, and providing accurate and reliable predictions, especially for NLP tasks containing thousands of concept features [25, 26].

With the different combinations among methods of concept extraction, vectorization, and ML models, we have 8 model variations, such as “BOW + TF-IDF + RF” and “CUI + TF-IDF + XGBoost.” As both methods, RF and XGBoost, use decision trees as the base models, we assigned 100 decision trees to them, respectively. These models’ performance was then estimated by fivefold cross validation, maintaining the same proportion of positive and negative patients in each group.

#### Performance metrics

To evaluate and compare the models developed, we calculated their sensitivity, specificity, positive predictive value (PPV), negative predictive value (NPV), and F1 score using chart data as a reference standard. We also calculated binomial proportional confidence intervals for all the metrics.

We compare the results with ICD-based CeVD identification algorithms in DAD after defining CeVD using ICD-10 codes (e.g., G45-46, I60-69, H34, see Additional file 1: Table S1) [3]. The performance metrics of the developed NLP models were reported on the same level of specificity as with the ICD-based algorithm.

## Results

### Characteristics of the study cohort

Among the 3036 patients, chart reviewers identified 360 patients with CeVD (see Table 1). Characteristics that were statistically significantly different (P < 0.05) between the CeVD positive cohort and negative cohort are: age, comorbidities such as atrial fibrillation, angina, hypertension, peripheral vascular disease (PVD) and obesity.

**Table 1:** Patients characteristics

Characteristics	All (percentage)	Patients with CEVD (percentage)	Patients without CEVD (percentage)	*P* value
N =	3036 (100%)	360 (11.9%)	2676 (88.1%)	
Demographic				
Median of age (IQR)	63.0 (48.9–76.5)	77.4 (67.0–85.9)	60.9 (46.4–74.2)	< 0.0001
Female	1528 (50.3%)	175 (48.6%)	1353(50.6%)	0.5
Comorbidities				
Atrial fibrillation	370 (12.2%)	106 (29.4%)	264 (9.9%)	< 0.0001
Angina	203 (6.7%)	41 (11.4%)	162 (6.1%)	0.0002
Myocardial infarction	102 (3.4%)	18 (5.0%)	84 (3.1%)	0.06
Hypertension	1469 (48.4%)	267 (74.2%)	1202 (44.9%)	< 0.0001
Peripheral vascular disease	148 (4.9%)	46 (12.8%)	102 (3.8%)	< 0.0001
Obesity	736 (24.2%)	68 (18.9%)	668 (25.0%)	0.01
Alcohol abuse	230 (7.6%)	19 (5.3%)	211 (7.9%)	0.08
Smoking	605 (19.9%)	66 (18.3%)	539 (20.1%)	0.4

*IQR* Interquartile range

### Characteristics of selected document types

We collected 49 types of clinical documents of patients during hospitalization, such as nursing transfer reports, inpatient consultations, and discharge summaries. The detailed text statistics for these document types can be found in Additional file 1: Table S2. For a better explanation, we consolidated these document types into 9 categories (see Additional file 1: Table S3).

Using the feedforward sequential selection, we identified four essential document types, “nursing transfer report,” “discharge summary,” “nursing notes,” and “inpatient consultation.” These documents are sensitive and informative for CeVD detection. Table 2 shows the statistics of patients, documents, and words. At least 90% of patients (with or without CeVD) have at least 2 types of documents. These four types of documents complement each other in providing sufficient clinical information to identify CeVD.

**Table 2:** Characteristics of extracted documents

Document type	All (n = 3036)	Patients with CeVD (n = 360)	Patients without CeVD (n = 2676)
Median number of notes per patient (IQR)	2.0 (1.0–2.0)	2.0 (1.0–2.0)	2.0 (1.0–2.0)
Number of patients with at least 2 types of documents (%)	2774 (91.4)	344 (95.6)	2430 (90.8)
Median word count per note (IQR)	430.0 (310.0–678.0)	434.5 (322.2–723.0)	428.0 (308.0–675.0)

Detailed document types: nursing transfer report—emergency department to inpatient, discharge summary-medical; surgical assessment and history, inpatient consultations, and discharge summary

**Fig. 3:**
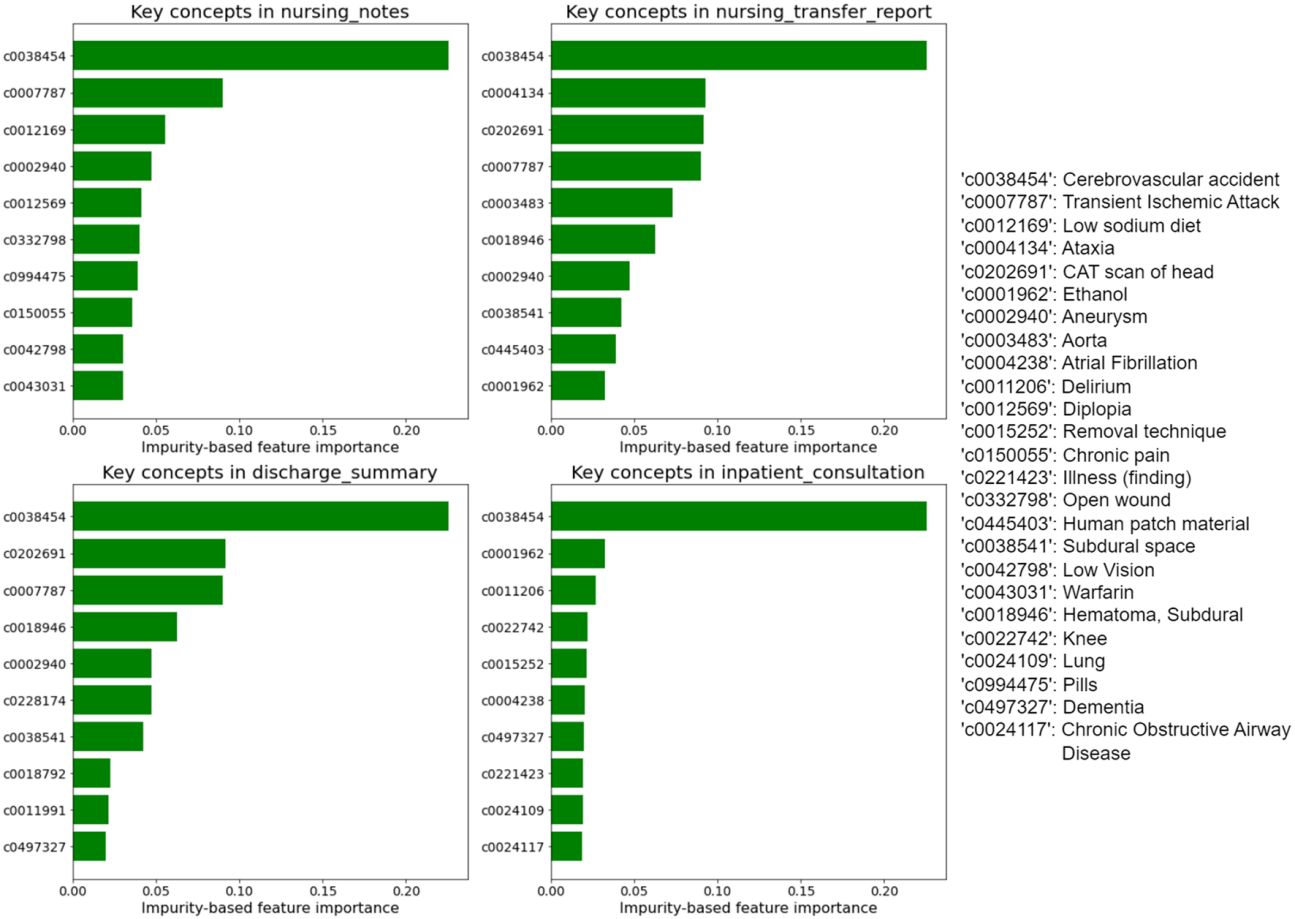
Top 10 key concepts for detecting CeVD in each selected document type. The concepts were UMLS terms extracted by cTAKES. The impurity-based feature importance measured the importance of classifying CeVD

To examine how these document types contribute to CeVD detection, the top ten key concepts in each document type were analyzed, as shown in Fig. 3. There are some common and vital concepts across four document types, such as “C0038454” (stroke-related concepts) and “C0007787” (transient ischemic attack). It is reasonable that the existence of these concepts can directly reflect the CeVD status. The remaining concepts are less overlapped and unique to each document type, such as “C0012169” (low sodium diet) in “nursing notes,” “C0004134” (ataxia) in “nursing transfer report,” “C0202691” (CAT scan of head) in “discharge summary,” and “C0001962” (ethanol) in “inpatient consultation.” This demonstrated that these document types contain essential concepts and can supplement each other to gain more comprehensive information in CeVD detection.

### Classification performance

The top 4 trained models were shown in Table 3. XGBoost generally outperformed the random forest method. TF-IDF performed better than term count when comparing models “CUI + word count + XGBoost” and “CUI + TF-IDF + XGBoost.” Similarly, the concept extraction method “CUI” had better performance than “Bag of Words (BOW).” Consequently, the combination of XGBoost, TF-IDF, and CUI achieved the best performance over other ML models in the metrics of sensitivity (70%), specificity (99.1%), PPV (87.8%), NPV (97.1%), F1 (77.8%), and accuracy (96.5%).

**Table 3:** CeVD case identification with DAD and EMR

Model	Sensitivity% (95% CI)	Specificity% (95% CI)	PPV% (95% CI)	NPV% (95% CI)	F1%	Accuracy% (95% CI)
ICD-10-CA-codes in DAD	25.0 (20.6–29.8)	**99.3 (98.9–99.6)**	82.6 (74.5–88.5)	90.8 (90.3–91.3)	38.4	90.5 (89.4–91.5)
CUI + TF-IDF + RF	65.8 (60.7–70.7)	98.5 (98.0–99.0)	85.9 (81.5–89.3)	95.5 (94.9–96.1)	74.1	94.7 (93.8–95.4)
CUI + word count + XGBoost	68.1 (63.0–72.8)	98.6 (98.1–99.0)	86.9 (82.7–90.2)	95.8 (95.2–96.4)	76.2	95.0 (94.2–95.7)
CUI + TF-IDF + XGBoost*	**70.00 (65.0–74.7)**	99.1 (98.7–99.3)	**87.8 (83.7–91.0)**	**97.1 (96.6–97.5)**	**77.8**	**96.5 (95.8–97.0)**
BOW + TF-IDF + XGBoost	59.2 (53.9–64.3)	98.7 (98.1–99.1)	85.5 (80.9–89.2)	94.7 (94.1–95.3)	69.4	94.0 (93.1–94.8)

The value in bold indicates the best among other approaches in that specific metric

We also compared the model performance with ICD-10-CA-based methods. With similar specificity (99.3% in ICD-10-CA vs 99.1% in model “CUI + TF-IDF + XGBoost”), the performance in other metrics is improved hugely by the obtained model, such as sensitivity increased from 25.0 to 70.0%, and F1 increased from 38.4% to 77.8%.

**Fig. 4:**
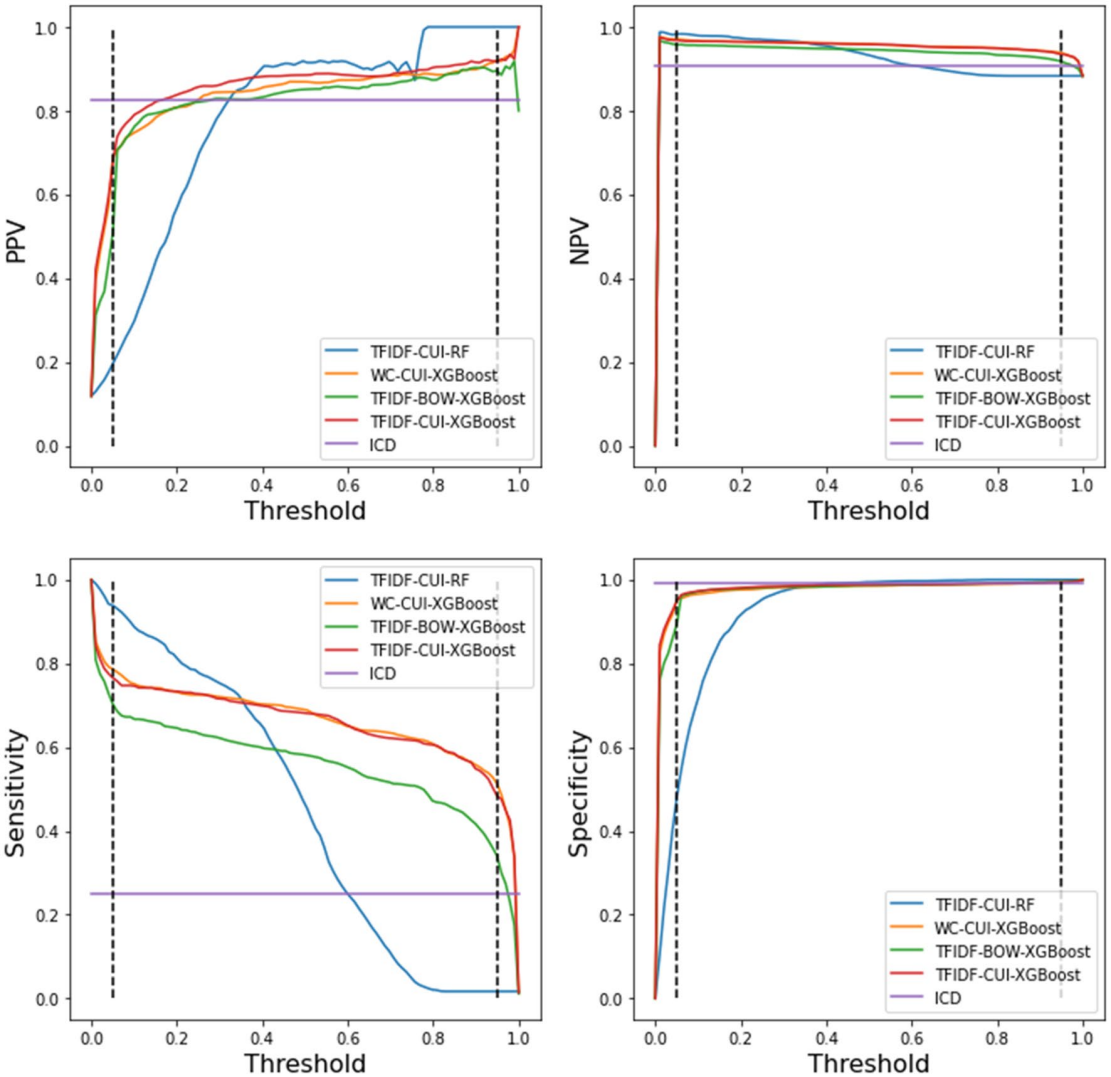
PPV, NPV, sensitivity, and specificity of the four NLP models and ICD algorithm, with changing thresholds ranging between 0.05 and 0.95. The two dashed lines in each subfigure represent the 0.05 and 0.95 threshold bounds, respectively. TFIDF-CUI-RF represents algorithm “CUI + TF-IDF + RF”; WC-CUI-XGBoost represents algorithm “CUI + word count + XGBoost”; TFIDF-BOW-XGBoost represents algorithm “BOW + TF-IDF + XGBoost”; TFIDF-CUI-XGBoost represents algorithm “CUI + TF-IDF + XGBoost”; ICD represents the ICD-10-CA-codes in DAD algorithms, respectively

We included the four metrics of the four NLP models with changing threshold values from 0.05 to 0.95, as shown in Fig. 4. Since the ICD algorithm is deterministic, its threshold is not changeable. The PPVs of “CUI + TF-IDF + RF,” “CUI + word count + XGBoost,” “BOW + TF-IDF + XGBoost,” and “CUI + TF-IDF + XGBoost” started to exceed the performance of ICD at thresholds 0.32, 0.25, 0.28, and 0.17 within the threshold bound, respectively. “CUI + word count + XGBoost” and “CUI + TF-IDF + XGBoost” had very similar and robust performance with the change of thresholds, whereas “CUI + TF-IDF + RF” was affected significantly. Generally, the “CUI + TF-IDF + XGBoost” algorithm achieved better and more robust performance with smaller thresholds.

## Discussion

This paper shows that EMR textual information abstracted by NLP techniques outperforms traditional ICD codes for assessing cerebrovascular disease, and compares favourably with resource-intensive chart review using a fraction of human resources. With the prevalence of 11.8% CeVD in over 3000 records, the developed NLP model significantly improves the validity of DAD-based ICD algorithm (sensitivity: 70% vs. 25% and PPV: 88% vs. 83%).

EMR data is more informative and efficient in identifying CeVD patients than conventionally used hospitalization data (i.e., DAD). First, due to the high volume of discharges, coders have limited time to code patients comprehensively, causing missing codes and low quality. Second, there is no uniform international definition of the most responsible diagnosis, which varies between the primary reason for admission and the condition with intensive resource usage [27]. When looking for conditions contributing primarily to the length of stay in hospital (a Canada-wide used definition), CeVD is likely under-coded as it can be a comorbidity causing admission. Conversely, EMRs contain many documents not usually used by medical coders. As identified in this study, four types of documents (i.e., “nursing transfer report,” “discharge summary,” “nursing notes,” and “inpatient consultation”) jointly contribute to the accurate detection of CeVD by providing more comprehensive medical information. Restricting the analysis to a specific document type, therefore, has the potential to impede detection.

To abstract the knowledge from these EMRs textual data, NLP techniques are essential. Information extraction from unstructured text is known to be difficult, and contains subtasks including NER, relation extraction, and pattern extraction. The text-based classification assigns categorical labels for a text fragment by finding the patterns composed of NERs and their relationships. By comparing different combinations of NLP models, we identified the optimal model, CUI + TF-IDF + XGBoost. The TF-IDF performs better than word count because it can efficiently eliminate low-sensitive concepts in differentiating positive and negative groups. CUI is a better concept extraction method than the NER by scispaCy because cTAKES can merge similar concepts into one, such as “stroke,” “CVA,” and “brain vascular accidents” are mapped to the same CUI “C0038454”. Sine XGBoost has better capability in dealing with overfitting and allows a more general model than random forest, it shows a slightly better performance in detecting CeVD, as shown in Table 3.

We recognized that there were new NLP techniques being applied to EMR data, such as transformer-based language model [28]. In our previous work on identifying pressure injuries using ClinicalBERT [29], the model did not outperform XGBoost and random forest algorithms due to the relatively small sample size. Without special treatments, such as fine-tuning of transformer-based language models, data augmentation for imbalanced classes, architecture tuning of neural networks, and cost-sensitive learning, the native deep learning model cannot perform well on the proposed identification task. Compared with deep learning models, XGBoost and random forest algorithms are easier to be deployed in local settings with better interpretability and less computational cost. We expect deep learning models to eventually have superior performance with the mentioned comprehensive investigations. Our additional works were concurrently conducted for the identification of CeVD and other conditions using transformer-based NLP techniques.

The widespread use of text based EMR algorithms to supplement ICD codes and traditional chart reviews has many potential advantages for epidemiology and health outcomes research. CeVD status is frequently used as an important factor in stratifying outcomes in population health research. While some outcomes, such as ischemic stroke, have reasonable validity, other aspects of CeVD, such as carotid atherosclerosis, are likely poorly coded. This probably explains the poor sensitivity (25%) of ICD codes for CeVD in our study. We achieved 88% PPV and 70% sensitivity, an improvement over the widely adopted ICD-based algorithm. Given the amount of knowledge contained in clinical text, the algorithm is applicable to detecting many other diseases, especially conditions with under-coding issues. Text based EMR algorithms may be used to periodically re-evaluate the validity of existing ICD code-based approaches and ensure that ICD code validity is not changing over time.

Although our findings demonstrate the superior performance compared to ICD codes, there is still ample room for further exploration into the comprehensive utilization of EMR data for CeVD case identification. The detailed stratifications of patients were often beneficial for producing tailored treatment strategies and inclusion or exclusion criteria in clinical trials [30]. To enable the appropriate stratification, many efforts were underway to phenotype subclasses of various CeVD categories, such as ischemic stroke [9, 10] and cerebral aneurysms [12]. Further NLP methods could facilitate the identification of other categories, such as hemorrhagic stroke and transient ischemic attack. The EMR data-based algorithms can be utilized to potentially examine underlying patterns and risk factors. Some researchers were using text data to analyze the causes of transient ischemic attack [13] and complications of ischemic stroke [14]. Additionally, these algorithms can be applied for real-time monitoring and surveillance of CeVD at a population level. Public health authorities can thus identify trends, assess disease burden, and implement targeted interventions. With demonstrated richness of knowledge in EMR data, NLP models can expedite and facilitate data usage for these purposes.

## Limitations

There are some limitations in this study. First, further examination of missing cases is needed, as 30% of cases are still missed by the proposed algorithm using EMR data. The missing cases are likely caused by variations in clinical documents and the capability of NLP models to detect them. We believe that the performance of the NLP models can be further improved by having better NER and incorporating sequential and contextual patterns among recognized concepts. Second, the data we studied is only from one city (i.e., Calgary). EMR diversities in format and content could be subject to change when larger populations and geographies are considered. The identified sensitive document types will vary accordingly. Then, we recognize that the time span of the study dataset is short, as they lie between January to June 2015. Therefore, the model might not account for variations or trends over a longer time period, such as seasonal variations in disease occurrence or changes in medical practice. Lastly, we did not validate the algorithms in external databases. We aim to collaborate with other institutions and collect data from multiple geographic regions to strengthen the external validity of our findings and encourage researchers to apply this method to their datasets for validation and improvement using our project publicly available on GitHub.^1^

## Conclusion

Compared to the widely used ICD-based algorithm, the EMR NLP model significantly improved the sensitivity and PPV while maintaining similar specificity. This algorithm could be used to enhance existing ICD databases, for health research and surveillance.

### Supplementary Information

**Additional file 1: Table S1. **ICD-10 codes to identify CeVD patients [3]. **Table S2.** Text statistics of various document types for patients with CeVD. **Table S3.** Consolidated document types. The 49 document types were merged into 9 categories.

## Data Availability

The data sets analyzed in this study are not publicly available due to the risk of exposing idenficiable information contained within the clinical notes. Access to the data is restricted to those collaborate with the Centre for Health Informatics and Alberta Health Services.

## Footnotes

1 https://github.com/jp-uofc/CeVD-phenotyping
