# Supplementary material for: Cerebrovascular disease case identification in inpatient electronic medical record data using natural language processing
Source: Brain Inform. 2023 Sep 2;10(1):22. doi: 10.1186/s40708-023-00203-w (PMC10474977; doi:10.1186/s40708-023-00203-w)
Supplement: Supplementary file 1 — Additional file 1: Table S1. ICD-10 codes to identify CeVD patients [3]. Table S2. Text statistics of various document types for patients with CeVD. Table S3. Consolidated document types. The 49 document types were merged into 9 categories. [file 40708_2023_203_MOESM1_ESM.docx]

# Additional file

Table S1 ICD-10 codes to identify CeVD patients [3].

| **Code** | **Meaning** |
| --- | --- |
| G45 | Transient cerebral ischemic attacks and related syndromes |
| G46 | Vascular syndromes of brain in cerebrovascular diseases |
| I60 | Nontraumatic subarachnoid hemorrhage |
| I61 | Nontraumatic intracerebral hemorrhage |
| I62 | Other and unspecified nontraumatic intracranial hemorrhage |
| I63 | Cerebral infarction |
| I64 | Stroke, not specified as hemorrhage or infarction |
| I65 | Occlusion and stenosis of precerebral arteries, not resulting in cerebral infarction |
| I66 | Occlusion and stenosis of cerebral arteries, not resulting in cerebral infarction |
| I67 | Other cerebrovascular diseases |
| I68 | Cerebrovascular disorders in diseases classified elsewhere |
| I69 | Sequelae of cerebrovascular disease |
| H34 | Retinal vascular occlusions |

1. **Preprocessing procedures of EMR text**

The detailed preprocessing in this study includes the following steps:

1. Data cleaning

This objective is to convert each patient’s note into a clean, normalized, and standardized format that facilitates the subsequent concept extraction. The specific tasks include lowercasing text, spelling check and correction, stemming and lemmatization, the removal of punctuation, numerical values, special characters, and stop words [31]. These tasks were standard procedures for text cleaning and can be completed by a Python package NLTK^[[1]](#footnote-1)^.

1. Concept extraction

The goal of this step is to extract concepts from the cleaned text. We used Named Entity Recognition (NER) to perform concept extraction. NER is a subtask of NLP that seeks to identify named objects from free-text. Named entities in clinical field include “ICU,” “Glasgow Coma Scale,” “hepatocellular carcinoma,” etc. We used two biomedical concept extraction methods:

1. ScispaCy

ScispaCy^[[2]](#footnote-2)^ is a Python package containing spaCy^[[3]](#footnote-3)^ models for processing biomedical, scientific or clinical text. ScispaCy provides NLP models more suitable for biomedical text because it is fully trained in the MedMentions^[[4]](#footnote-4)^ dataset, an annotated 4392 abstracts of scientific papers released in PubMed.

1. cTAKES

We processed all the clinical notes using the clinical Text Analysis and Knowledge Extraction System (cTAKES)^[[5]](#footnote-5)^. We extracted clinical concepts from UMLS^[[6]](#footnote-6)^ in the form of CUI. This approach accounts for the variation in terminology between EMRs, as UMLS maps synonyms to the same underlying concepts. For example, in the UMLS, the clinical concept “cerebrovascular accident” is designated as CUI “C0038454”. Vocabularies with the same semantic meaning of cerebrovascular accident, such as “stroke,” “CVA,” and “brain vascular accidents,” are mapped to the same CUI “C0038454”. Therefore, using cTAKES to extract CUI gives us consistent features. We also leveraged cTAKES negation detection and topic attribute annotators to remove negated concepts (e.g., no complications, did not find evidence of …) and CUI associated with someone else but not the patient.

We compared two concept extraction methods for clinical fields in the article. Both methods can identify the clinical concepts from the cleaned text.

1. Vectorization

The vectorization is to transform the extracted concepts into numerical representations suitable for machine learning algorithms to conduct CeVD classification. We used BOW method to extract features from patient text documents by turning the concepts into numeric values located at specific indices of vector. Each concept can be scored by two strategies in this study: word count and term frequency-inverse document frequency (TF-IDF), by Scikit-learns^[[7]](#footnote-7)^ counting vectorizer and Term Frequency-Inverse Document Frequency vectorizer. Eventually, the note was represented by a BOW vector that can serve as the input for machine learning algorithms.

We transformed each patient’s selected document into a BOW vector using the above procedure and paired all the vectors with the presence status of CeVD as the training data for XGBoost and random forest model learning.

1. **Document Type Statistics**

Table S2 Text statistics of various document types for patients with CeVD.

| **Document type** | **#Words (STD)** | **#Sentences (STD)** | **#Patients** |
| --- | --- | --- | --- |
| MPR | 124.7 (131.1) | 10.1 (9.5) | 360 |
| Clinical Record | 105.5 (56.4) | 3.6 (3.3) | 356 |
| Pain Assessment | 39.7 (17.2) | 2.6 (1.2) | 355 |
| Patient Care | 151.0 (50.7) | 20.3 (12.7) | 353 |
| Intake and Output | 18.8 (12.1) | 7.1 (2.9) | 319 |
| Adult Triage Note | 212.8 (29.4) | 8.4 (3.3) | 296 |
| Patient Assessment | 294.7 (109.4) | 47.7 (20.6) | 287 |
| Nursing Transfer Report - ED to IP | 343.1 (91.6) | 20.2 (9.5) | 271 |
| AcuityPlus Inpatient Classification | 54.1 (17.6) | 9.4 (1.5) | 227 |
| Discharge Summary - Medical | 965.4 (576.3) | 67.9 (60.0) | 217 |
| Patient Assessment Tools | 142.4 (30.8) | 17.5 (3.0) | 216 |
| Med Surg MET/NOT-MET Assessment | 192.6 (85.3) | 20.8 (5.7) | 198 |
| Med Surg MET/NOT-MET Assessment Flowsheet | 158.3 (92.1) | 17.7 (8.0) | 198 |
| ED Physician Handover Report | 84.97 (40.34) | 8.9 (5.1) | 156 |
| ED UCC - Intake and Output | 12.0 (6.2) | 5.9 (1.6) | 129 |
| Neurological Observation | 99.6 (9.2) | 14.7 (1.4) | 126 |
| Pharmacy Care Plan | 787.9 (701.0) | 48.3 (49.3) | 122 |
| Surgical Assessment and History - Nursing | 501.8 (159.8) | 8.7 (6.4) | 86 |
| Inpatient Operative/Procedure Report | 628.0 (290.6) | 47.4 (22.1) | 83 |
| Nursing Transfer Report - IP to IP | 517.6 (125.8) | 21.9 (8.7) | 72 |
| Patient Assessment Neuro | 403.6 (106.0) | 54.5 (29.3) | 68 |
| Nursing Transfer Report - PACU to IP | 254.9 (78.8) | 11.5 (7.3) | 66 |
| History and Physical | 732.4 (190.6) | 86.4 (28.9) | 46 |
| Discharge Summary | 425.0 (217.6) | 49.3 (30.8) | 45 |
| History & Physical Examination. | 1021.3 (526.9) | 74.9 (45.6) | 36 |
| Discharge Summary - General | 1036.5 (544.5) | 73.4 (42.0) | 33 |
| Inpatient Consultation | 791.4 (329.21) | 61.6 (31.1) | 31 |
| Inpatient Consult Report. | 1423.0 (887.2) | 112.9 (80.5) | 28 |
| Discharge Summary - Surgery Short | 308.4 (169.2) | 20.7 (13.6) | 27 |
| EEG Preliminary Report | 102.4 (42.3) | 7.7 (3.7) | 21 |
| Transfer Summary. | 652.8 (240.5) | 67.7 (32.2) | 13 |
| Outpatient Consultation | 755.2 (273.4) | 85.7 (37.8) | 12 |
| Acute Pain Service Summary Note | 493.2 (299.9) | 58.8 (47.1) | 10 |
| PICC / Midline Record | 245.1 (46.4) | 40.8 (16.3) | 7 |
| Neurological Diagnostics | 315.0 (71.7) | 32.2 (4.2) | 5 |
| Neuro Rehabilitation | 184.1 (227.3) | 31.67 (35.1) | 3 |
| Admitting Trauma History and Physical Assessment | 419.5 (2.5) | 15.0 (6.0) | 2 |
| Urological Diagnostics | 258.5 (16.5) | 19.0 (2.0) | 2 |
| Day Surgery / 24 Hour | 97.6 (33.4) | 5.0 (4.5) | 2 |
| Discharge Summary - Orthopedic Surgery | 191.5 (22.5) | 14.5 (3.5) | 2 |
| Social Work Assessment | 859.0 (0.0) | 65.0 (0.0) | 1 |
| Discharge Summary - Stroke Neurology | 336.0 (0.0) | 17.0 (0.0) | 1 |
| Mental Health MET/NOT-MET Assessment Flowsheet | 23.0 (2.0) | 6.5 (0.5) | 1 |
| AcuityPlus Mental Health Patient Classification | 31.0 (2.2) | 7.7 (0.5) | 1 |
| Nursing Transfer Report - Mental Health | 216.0 (0.0) | 10.0 (0.0) | 1 |
| History & Physical Examination | 681.0 (0.0) | 85.0 (0.0) | 1 |
| Mental Health MET/NOT-MET Assessment | 101.5 (8.5) | 17.5 (1.5) | 1 |
| Discharge Summary Thoracic Surgery | 1123.0 (0.0) | 81.0 (0.0) | 1 |

#words: the number of words contained in each document; #sentences: the number of sentences contained in each document; #patiens: the number of patients with CeVD having the specific document.

Table S3 Consolidated document types. The 49 document types were merged into 9 categories.

| **Document type** | **Consolidated Document Type** |
| --- | --- |
| Adult Triage Note | Adult Triage Note |
| Discharge Summary - Medical | Discharge Summary |
| Discharge Summary | Discharge Summary |
| Discharge Summary - General | Discharge Summary |
| Discharge Summary - Surgery Short | Discharge Summary |
| Discharge Summary - Orthopedic Surgery | Discharge Summary |
| Discharge Summary - Stroke Neurology | Discharge Summary |
| Discharge Summary Thoracic Surgery | Discharge Summary |
| Transfer Summary. | Discharge Summary |
| ED Physician Handover Report | Ed Physician Notes |
| History and Physical | History & Physical Examination |
| History & Physical Examination. | History & Physical Examination |
| Admitting Trauma History and Physical Assessment | History & Physical Examination |
| History & Physical Examination | History & Physical Examination |
| Inpatient Consult Report. | Inpatient Consult Report. |
| Inpatient Consultation | Inpatient Consultation |
| Acute Pain Service Summary Note | Inpatient Consultation |
| Inpatient Operative/Procedure Report | Inpatient Operative/Procedure Report |
| Med Surg MET/NOT-MET Assessment | Med Surg MET/NOT-MET Assessment |
| Med Surg MET/NOT-MET Assessment Flowsheet | Med Surg MET/NOT-MET Assessment |
| Mental Health MET/NOT-MET Assessment Flowsheet | Med Surg MET/NOT-MET Assessment |
| Mental Health MET/NOT-MET Assessment | Med Surg MET/NOT-MET Assessment |
| EEG Preliminary Report | Neurological Diagnostics |
| Neurological Diagnostics | Neurological Diagnostics |
| MPR - Multidisciplinary Progress Record | Non-physician Progress Notes |
| Clinical Record | Nursing Notes |
| Pain Assessment | Nursing Notes |
| Patient Care | Nursing Notes |
| Intake and Output | Nursing Notes |
| Patient Assessment | Nursing Notes |
| AcuityPlus Inpatient Classification | Nursing Notes |
| Patient Assessment Tools | Nursing Notes |
| ED UCC - Intake and Output | Nursing Notes |
| Neurological Observation | Nursing Notes |
| Surgical Assessment and History - Nursing | Nursing Notes |
| Patient Assessment Neuro | Nursing Notes |
| Day Surgery / 24 Hour | Nursing Notes |
| AcuityPlus Mental Health Patient Classification | Nursing Notes |
| Nursing Transfer Report - ED to IP | Nursing Transfer Report |
| Nursing Transfer Report - IP to IP | Nursing Transfer Report |
| Nursing Transfer Report - PACU to IP | Nursing Transfer Report |
| Nursing Transfer Report - Mental Health | Nursing Transfer Report |
| Outpatient Consultation | Outpatient Consultation |
| Pharmacy Care Plan | Pharmacy Care Plan |
| PICC / Midline Record | PICC / Midline Record |
| Neuro Rehabilitation | Rehabilitation |
| Social Work Assessment | Social Work Assessment |
| Urological Diagnostics | Urological Diagnostics |

1. https://www.nltk.org/ [↑](#footnote-ref-1)
2. <https://spacy.io/universe/project/scispacy> [↑](#footnote-ref-2)
3. <https://spacy.io/> [↑](#footnote-ref-3)
4. <https://github.com/chanzuckerberg/MedMentions> [↑](#footnote-ref-4)
5. <https://ctakes.apache.org/> [↑](#footnote-ref-5)
6. <https://www.nlm.nih.gov/research/umls/index.html> [↑](#footnote-ref-6)
7. https://scikit-learn.org/stable/ [↑](#footnote-ref-7)
